# The Use of Mid-Pregnancy Cervical Length to Predict Preterm Birth in Brazilian Asymptomatic Twin Gestations

**DOI:** 10.1055/s-0043-1769467

**Published:** 2023-05-24

**Authors:** Thaís Valéria Silva, Anderson Borovac-Pinheiro, Marcelo Santucci França, Kaline Fernandes Marquat, Juliana Passos Argenton, Ben Willem Mol, Rodolfo Carvalho Pacagnella

**Affiliations:** 1 Department of Obstetrics and Gynecology, School of Medicine, University of Campinas, Campinas, SP, Brazil; 2 CISAM Maternity Hospital, University of Pernambuco, Recife, PE, Brazil; 3 Department of Obstetrics, Screening and Prevention of Preterm Birth Sector, Discipline of Fetal Medicine, Escola Paulista de Medicina, Federal University of Sao Paulo, São Paulo, SP, Brazil; 4 Department of Obstetrics and Gynaecologic, Monash University, Clayton, Victoria, Australia; 5 Aberdeen Centre for Women's Health Research, University of Aberdeen, Aberdeen, United Kingdom

**Keywords:** Cervical length measurement, Preterm birth, Prematurity, Multiple pregnancy, Medida do colo uterino, Parto prematuro, Prematuridade Gestação gemelar

## Abstract

**Objective** To describe a reference curve for cervical length (CL) in mid-trimester twin gestations using transvaginal ultrasound (TVU) and to investigate whether short CL increases spontaneous preterm birth (sPTB) in asymptomatic twin pregnancies.

**Methods** This was a prospective cohort study performed at 17 outpatient antenatal facilities of Brazil with women at 18 0/7 to 22 6/7 weeks of gestation who participated in a randomized clinical trial screening phase (P5 trial) between July 2015 and March 2019. TVU was performed to provide CL measurement in all screened women. Almost all women with CL ≤ 30 mm received vaginal progesterone 200mg/day and they were also randomized to receive cervical pessary or not. We considered data from the CL distribution among asymptomatic twin pregnancies and analyzed CL and its association with PTB generating receiver operating characteristics (ROC) curves and Kaplan-Meier curves.

**Results** A total of 253 pregnant women with twins were included in the distribution curve. The mean CL was 33.7 mm and median was 35.5mm. The 10th percentile was 17.8mm. We identified a PTB rate of 73.9% (187/253) with 33.6% of sPTB < 37 (85/253) and 15% (38/253) of sPTB < 34 weeks. The best cutoff point to predict sPTB < 37 was 24.15 mm. However, the ROC curve showed a poor performance (0.64). The Kaplan-Meier survival curves identified that only CL values ≤ 20mm were associated to sPTB < 34 weeks.

**Conclusion** A cutoff point of CL ≤ 20 mm can be interesting point to identify short cervix in Brazilian twin pregnancies. However, in Brazilian asymptomatic twin pregnancies, CL does not show a good performance to predict PTB.

## Resumo

**Objetivo** Descrever uma curva de referência da medida do colo uterino no Segundo trimestre de gestações gemelares através de ultrassonografia transvaginal (TVU) e investigar a correlação entre a medida do colo uterino (CL) e o parto prematuro espontâneo (sPTB) em pacientes assintomáticas.

**Métodos** Foi realizado uma coorte prospectiva multicêntrica em 17 centros de referência do Brasil com mulheres com gestação gemelar entre 18 0/7 a 22 6/7 semanas de gestação que participaram da primeira fase de um ensaio clínico randomizado (P5 trial) entre Julho/2015 a Março/2019. TVU foi realizada para obter a medida do colo uterino em todas as mulheres. A maioria das mulheres com CL ≤30 mm receberam progesterona por via vaginal 200mg/dia e estas foram randomizadas para receber ou não um pessário cervical. Este estudo considerou dados da medida do colo uterino entre mulheres assintomáticas, desenvolvendo uma curva de referência para gestantes gemelares e sua capacidade de predição do parto prematuro através de curva ROC (receiver operating characteristics) e curvas de sobrevida de Kaplan-Meyer.

**Resultados** O total de 253 gestantes foram incluídos no estudo, A média do CL foi 33.7mm e a mediana 35.5mm. O Percentil 10 do CL foi 17.8mm. A taxa de parto prematuro foi de 73.9% (187/253) com 33.6% de sPTB < 37 (85/253) e 15% (38/253) de sPTB < 34 semanas. O melhor ponto de corte para predizer sPTB < 37 foi 24.15 mm, entretanto a curva ROC demonstrou baixa performance (0.64). A curva de Kaplan-Meier para sPTB identificou que apenas CL ≤ 20 mm estavam associados a sPTB < 34 semanas.

**Conclusão** Colo uterino ≤20 mm pode ser um interessante ponto de corte para identificar colo curto entre gestações gemelares assintomáticas brasileiras. Entretanto, a medida do colo uterino não apresentou boa performance para predizer parto prematuro.

## Introduction

Multiple gestations are at higher risk for preterm birth (PTB), and neonatal morbidity and mortality, and their incidence has been in a rising trend since many of them are associated with assisted reproduction treatments. 1 2 3 The largest cohort in Brazil focused on risk factors for prematurity demonstrated that twin pregnancies had higher chance of PTB than singletons (OR: 15.61; 95% confidence interval, CI: 6.24–39.04). 4

The use of risk factors to identify women at higher risk of preterm delivery is part of the prevention strategies. 5 6 In this scenario, the cervical length (CL) measurement by transvaginal ultrasound (TVU) in singleton mid-trimester pregnancies has an important role to estimate the risk for spontaneous preterm birth (sPTB) associated with a short cervix. 7

Studies involving singleton pregnancies have considered 25 mm as the most accepted cutoff to define a short cervix, 8 which represents women under the 10 ^th^ percentile of an international reference curve. 7 Following this rationale, studies involving twin pregnancies also have focused on CL ≤ 25 mm as a short cervix. 9 10 However, this inference is highly questioned, since singleton and twin births present different CL distribution curves, 11 as well as different gestational outcome results. 2 So far, there is no consensus about the best cutoff point to define a short CL for twins, which makes clinical practice decisions regarding therapies for multiple gestations with short cervix even more difficult. 12

To correctly identify the CL that is associated to sPTB, specific populational distribution curves are necessary to describe the range of CL and to suggest what should be considered a short cervix in Brazilian twins' pregnancies. Moreover, it is important to know if it is possible to use CL as a predictor for sPTB in twin gestations. The main objective of this study was to describe a reference curve for CL in mid-trimester twin gestations using TVU and to identify the association between CL and gestational age at birth, and whether mid-pregnancy CL is a good predictor for PTB.

## Methods

We performed an ancillary analysis using a cohort strategy analysis of all twin pregnancies included in the P5 Trial (Pessary Plus Progesterone to Prevent Preterm Birth Study – Trial registration RBR-3t8prz, approved by the Brazilian National Review Board/CONEP – number 1.055.555) to describe Brazilian populational curves. 13

The P5 Trial was a multicenter, randomized, controlled trial involving 17 institutions (nine states in three regions: South, Southeast, and Northeast of Brazil) that compared the effectiveness of vaginal progesterone alone versus progesterone plus cervical pessary in women with short cervix. The study was coordinated by the University of Campinas from July 2015 to March 2019. A TVU screening program using a GE Logiq C5 (GE HealthCare. Chicago, IL, EUA) equipment or similar with a 5 to 9MHz transvaginal probe was offered as part of standard care for all women attending the ultrasound department during routine second trimester ultrasonographic examinations between 18 0/7 and 22 6/7 weeks. Women received information about the TVU technique and P5 study and all provided written informed consent. Sociodemographic characteristics, obstetric history, and current pregnancy information were previously collected.

Exclusion criteria for CL measurement were related to symptoms or pregnancy complications: painful contractions, vaginal bleeding, cerclage during current pregnancy before the screening, ruptured membranes diagnosed before screening, severe liver disease, cholestasis during this pregnancy, previous or current thromboembolism, placenta previa, cervical dilation greater than 1 cm, monoamniotic twin pregnancy, higher order multiple pregnancies (triplets or higher), major fetal malformation in at least one fetus, and stillbirth. For this analysis, we also excluded singleton gestations. The information on pregnancies was accessed using an online database from the screening phase of the P5 trial. Considering P5 trial interventions, 80 women had CL ≤ 30mm and 71 accepted to participate in the RCT, where 71 of the patients received progesterone and 43 also received a cervical pessary.

All participating sonographers received previous training in CL measurement according to the Fetal Medicine Foundation program, as well as additional training regarding the volume measurement developed by the University of Campinas's ultrasound department.

Describing the TVU technique briefly, after emptying the bladder, the woman was placed in the dorsal lithotomy position. The TVU probe was introduced until the anterior fornix region, avoiding extra pressure on the cervix, which can artificially increase the CL. A sagittal view of the cervix, showing the endocervical mucosa, was used to properly identify the internal and external ostium (os). Sludge and funneling were also evaluated and described, if present.

Descriptive statistical analysis was performed for demographic characteristics, expressed as means and percentages. Logistic regression was used to estimate odds ratio (OR) for baseline characteristics, gestational age, and CL measurements. Mean, median, and percentiles of CL (P5, P10, P25, P50, P75, P90, and P95) were obtained for the descriptive analysis. The receiver operating characteristics (ROC) curve analysis was performed to identify the most effective cutoff point to predict a sPTB (< 37 weeks). We also used the ROC curve analysis to identify the most effective cutoff points to predict overall PTB (< 37) and sPTB at different gestational ages (< 37 and > 34 weeks – later PTB; < 34–PTB; and < 28 weeks – extreme PTB). We calculated sensitivity, specificity, negative (NPV) and positive predictive values (PPV), and likelihood ratios (LR). The Kaplan-Meyer survival curves were used to analyze time to delivery, considering CL intervals (≤ 10 mm, 10–15 mm, 15–20 mm, 20–25 mm, 25–30 mm, 30–35 mm, 35–40 mm, and > 40 mm). A *p¬* -value < 0.05 was considered as statistically significant. All statistical analyses were performed using the R (R Foundation for Statistical Computing, Vienna, Austria) software, version 3.6.2.

## Results

**Fig. 1:**
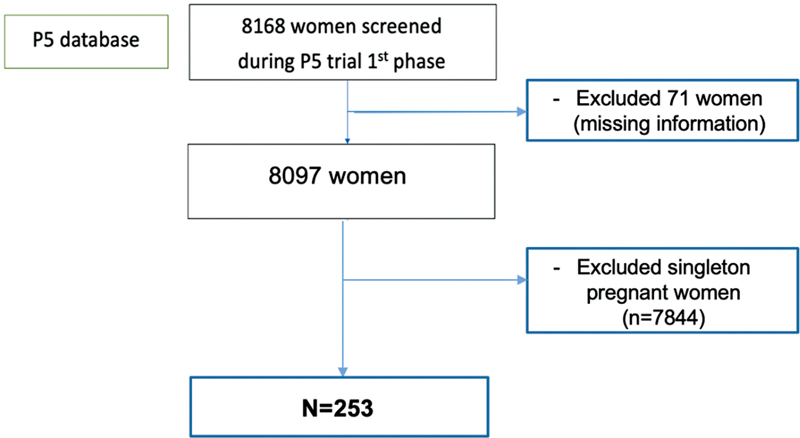
Patient enrolment flowchart.

A total of 253 from 8,168 women were included in this analysis. We excluded 71 women due to missing information, and 7844 singleton women ( Fig. 1 ). Women with CL ≤ 30 mm received progesterone 200 mg/day (71 women) and part of them also received a cervical pessary (28).

The incidence of twin pregnancy in the P5 screening phase was 3.5%, with 157 dichorionic diamniotic twins (62%), and 96 monochorionic diamniotic twins (38%). Approximately 70.8% (179/253) of the women were between 20 and 34 years old, and 86.9% (220/253) had studied less than 11 years. Most of the women, 53.4% (135/253), were non-white, and 32.8% (83/253) were obese (body mass index, BMI > 30). Considering obstetrical history, 58.5% (148/253) had at least one previous pregnancy, 7.5% (19/253) had at least one previous PTB, and 24.9% (63/253) had a previous abortion. Funneling was present in 10.7% (27/253), and 13.4% (34/253) presented sludge at measurement. The rate of PTB was 73.9% (187/253), with 33.6% of sPTB < 37 weeks (85/253), and 15% (38/253) of sPTB < 34 weeks ( Table 1 ).

**Table 1:** Sociodemographic and baseline characteristics x gestational age at birth

	Total ( *n* = 253)		Overall PTB < 37		≥ 37 weeks		OR (95% CI)	Spontaneous PTB < 37		≥ 37 weeks		OR (95% CI)	Spontaneous PTB < 34		≥ 34 weeks		OR (95% CI)
Characteristics	n or Mean	% or SD	n or Mean	% or ± SD	n or Mean	% or ± SD		n or Mean	% or ± SD	n or Mean	% or ± SD		n or Mean	% or ± SD	n or Mean	% or ± SD	
**Maternal age at measurement (years)**				±		±			±		±			±		±	
≤19	21	8.3	15	8	6	9.1	0.84 (0.32–2.47)	11	12.9	6	9.1	1.37 (0.49–4.25)	5	13.2	14	7.5	1.96 (0.59–5.68)
20–≤34	179	70.8	134	71.7	45	68.2		60	70.6	45	68.2		24	63.2	132	71	
> 35	53	20.9	38	20.3	15	22.7	0.85 (0.43–1.73)	14	16.5	15	22.7	0.7 (0.3–1.6)	9	23.7	40	21.5	1.24 (0.51–2.8)
** BMI (kg/m ^2^ ) **																	
≤18.5	4	1.6	4	2.1	0		NS	2	2.3	0	0	NS	2	5.3	2	1.1	NS
18.5–25	77	30.4	63	33.7	14	21.2		34	40	14	21.2		14	36.8	52	28	
25–30	89	35.2	59	31.6	30	45.5	0.44 (0.21–0.89)	25	29.4	30	45.5	0.34 (0.15–0.77)	12	31.6	68	36.6	0.66 (0.28–1.54)
> 30	83	32.8	61	32.6	22	33.3	0.62 (0.28–1.3)	24	28.2	22	33.3	0.45 (0.19–1.04)	10	26.3	64	34.4	0.58 (0.23–1.4)
**Ethnic origin (self-reported)**																	
Non-white	135	53.4	96	51.3	39	59.1		53	62.4	39	59.1		21	55.3	102	54.8	
White	118	46.6	91	48.7	27	40.9	1.37 (0.78–2.43)	32	37.6	27	40.9	0.91 (0.47–1.76)	17	44.7	84	45.2	1.11 (0.54–2.28)
**Schooling**																	
Preschool, Elementary	163	64.4	123	65.8	40	60.6		56	65.9	40	60.6		27	71.1	116	62.4	
Middle school	57	22.5	38	20.3	19	28.8	0.65 (0.34–1.27)	19	22.4	19	10.6	0.74 (0.35–1.58)	6	15.8	45	24.2	0.61 (0.21–1.5)
Highschool and Higher education	33	13	26	13.9	7	10.6	1.21 (0.51–3.21)	10	11.8	7	13	1.06 (0.37–3.14)	5	13.2	25	13.4	0.91 (0.29–2.45)
**Comorbidities**																	
No comorbidities	201	79.4	147	78.6	54	81.8		71	83.5	54	81.8		36	94.7	148	79.6	
Hypertension	16	6.3	12	6.4	4	6.1	1.1 (0.37–4.07)	7	8.2	4	6.1	1.37 (0.39–5.45)	0		13	7	NS
Endocrinopathies ^a^	14	5.5	10	5.3	4	6.1	0.92 (0.29–3.46)	3	3.5	4	6.1	0.59 (0.11–2.77)	1	2.6	10	5.4	0.43 (0.02–2.36)
Others ^b^	22	8.7	18	9.6	4	6.1	1.65 (0.59–5.92)	4	4.7	4	6.1	0.78 (0.18–3.45)	1	2.6	15	8.1	0.29 (0.02–1.49)
Previous conization (yes)	3	1.2	3	1.6	0		NS	0		0		NS	0	2.6	3	1.6	NS
**Obstetrical history**																	
Nulliparous	105	41.5	77	41.2	28	42.4		34	40	28	42.4		14	36.8	74	39.8	
Parous with no previous PTB	129	51	93	49.7	36	54.5	0.94 (0.52–1.67)	38	44.7	36	54.5	0.92 (0.47–1.83)	16	42.1	102	54.8	0.96 (0.43–2.2)
Parous with at least one previous PTB	19	7.5	17	9.1	2	3	3.09 (0.81–20.28)	13	15.3	2	3	5.69 (1.41–38.41)	8	21.1	10	5.4	4.87 (1.58–15.01)
Previous abortion (yes)	63	24.9	43	23	20	30.3	0.69 (0.37–1.3)	19	22.4	20	30.3	0.64 (0.3–1.34)	7	18.4	48	25.8	0.58 (0.21–1.4)
Funneling at measurement	27	10.7	24	12.8	3	4.5	3.09 (0.99–13.33)	15	17.6	3	4.5	3.9 (1.19–17.58)	9	23.7	14	7.5	2.93 (1.04–7.7)

**Abbreviations:** BMI, body mass index; CI, confidence interval; OR, odds ratio; PTB, preterm birth; NS, not significant. **Notes:** ^a^ Diabetes Mellitus, gestational diabetes, thyroidopathy. ^b^ Asthma, autoimmune diseases, anemia, obesity, hepatitis.

**Fig. 2:**
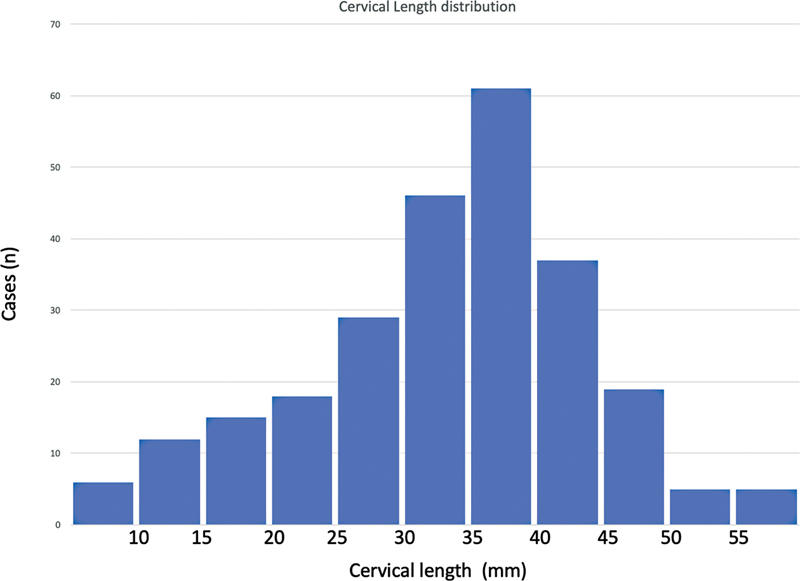
Cervical length (mm) distribution between 18 and 22 weeks gestation.

The univariate logistic regression analysis for PTB at < 37 weeks did not identify specifically risk factors ( Table 1 ). There was a trend to protection for PTB and sPTB < 37 among overweight and obese women. When considering only sPTB, we identified that having a previous PTB was a risk factor for sPTB < 37 (OR: 5.69; 95% CI: 1.41–38.41) and sPTB < 34 weeks (OR: 4.87; 95% CI: 1.58–15.01). Moreover, funneling at measurement was associated with sPTB < 37 (OR: 3.9; 95% CI: 1.19–17.58) and sPTB < 34 weeks (OR: 2.93; 95% CI: 1.04–7.7). The mean CL was 33.7 mm, and the median was 35.4 mm. The CL was ≤ 25 mm in 51 women (20.2%), ≤ 20 mm in 33 (13%), ≤ 15 mm in 18 (7.1%), and ≤ 10 mm in 6 (2.4%). The CL percentiles were P5 = 12.7 mm, P10 = 17.8 mm, P25 = 27 mm, P50 = 35.4 mm, P75 = 40.7 mm, P90 = 46 mm, and P95 = 48.3 mm. The CL at measurement showed a non-normal distribution confirmed by the Shapiro-Wilk test ( *p* < 0.001) ( Fig. 2 ).

Considering gestational age at measurement, there was a decrease in CL measure when gestational age increases ( Table 2 ).

**Table 2:** Values of percentile 5, 10, 25, 50, 75, 90, and 95 for the cervical length according to gestational age at measurement

Gestational age (weeks)	n	Mean	P05	P10	P25	P50	P75	P90	P95
Total	253	33.7	12.7	17.8	27	35.4	40.7	46	48.3
18	34	33.2	19.5	22.3	27.7	33.5	38.9	40	43.4
19	33	34.8	20.2	25.1	31.7	36.5	40	43	45.2
20	45	34.6	13.6	19.7	29	36.4	41.5	47.2	50.1
21	72	35.4	11.6	17.8	30.4	38	42.7	47	50.9
22	69	31.1	12.2	16.6	24.3	31.1	39.7	44.5	46

As a presumable consequence, considering two gestational ages intervals at measurement (18–20 vs. 21–22 weeks), we identified an increase in sensitivity to predict sPTB < 37 during 21 to 22 weeks ( Table 3 ).

**Table 3:** TVU accuracy to predict sPTB < 37 considering gestational age intervals

Measurement	Cervix at 18–20 weeks				Cervix at 21–22 weeks			
	≤ 24.15 mm	≤ 15 mm	≤ 25 mm	≤ 30 mm	≤ 24.15 mm	≤ 15 mm	≤ 25 mm	≤ 30 mm
Sensitivity	15	10	15	30	42.2	20	44.4	60
Specificity	100	100	93.5	83.9	91.4	100	82.9	74.3
PPV	100	100	75	70.6	86.3	100	76.9	75
NPV	47.7	46.2	46	48.1	55.1	49.9	53.7	59.1
Positive Likelihood Ratio	–	–	2.3	1.7	4.9	–	2.6	2.3
Negative Likelihood Ratio	0.9	0.9	0.9	0.8	0.6	0.8	0.7	0.5

**Abbreviations:** NPV, negative predictive values; PPV, positive predictive values, sPTB, spontaneous preterm birth, TVU, transvaginal ultrasound.

**Fig. 3:**
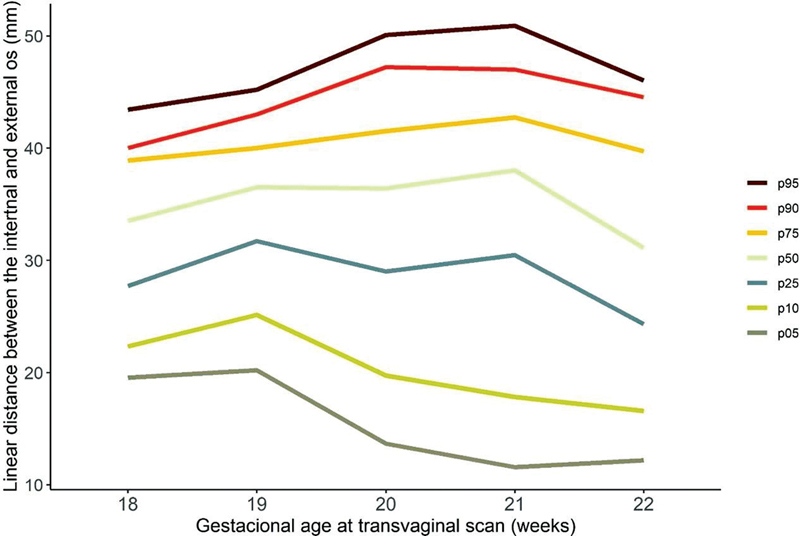
Curve of percentile values for CL measurement.

Fig. 3 illustrates the descriptive analysis of CL considering percentiles and gestational age at measurement.

**Fig. 4:**
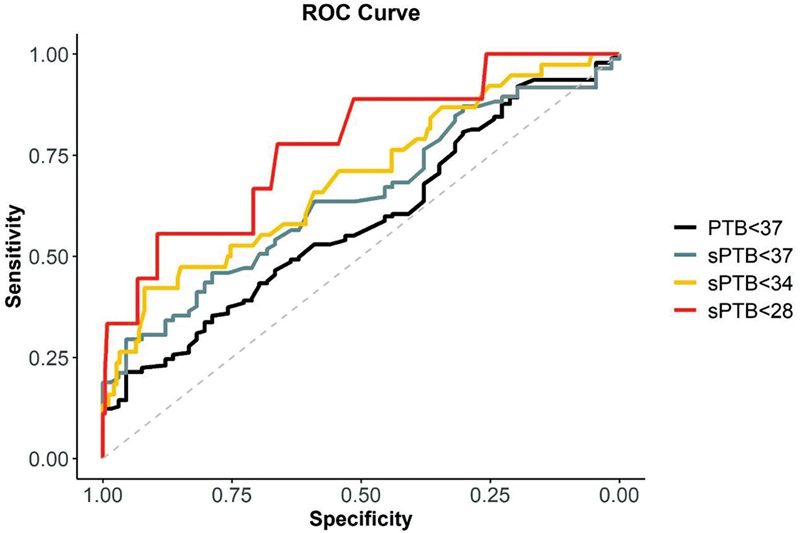
The ROC curve analysis of PTB and sPTB at different gestational ages.

The ROC curve analysis to predict sPTB at < 37 and < 34 weeks demonstrated a low performance, with area under the curve (AUC) 0.64 (0.56–0.73) and 0.69 (0.59–0.79), respectively. For sPTB at < 28 weeks the ROC curve demonstrated an AUC of 0.78 (0.60–0.95) ( Fig. 4 and Table 4 ). Table 4 illustrates CL performance tests results to predict prematurity. The best cutoff point to predict sPTB at < 37 weeks was 24.15 mm, with 24.9% sensitivity and 95.5% specificity.

**Table 4:** Cervical length performance for predicting PTB

	AUC	95% CI	*Cut-off*	Sensitivity	Specificity	PPV	NPV	LR+	LR-
PTB < 37	0.586	(0.509–0.663)	24.15	21.4%	95.5%	93.0%	30.0%	4.7	0.8
sPTB < 37	0.644	(0.557–0.732)	24.15	24.9%	95.5%	89.3%	51.2%	6.5	0.7
sPTB < 34	0.692	(0.594–0.79)	21.90	42.1%	91.9%	51.6%	88.6%	5.2	0.6
sPTB < 28	0.776	(0.607–0.946)	19.95	55.6%	89.6%	16.7%	98.1%	5.3	0.5

**Abbreviations:** AUC, area under the curve; CI, confidence interval; LR, likelihood ratios; NPV, negative predictive values; PPV, positive predictive values, PTB, preterm birth; sPTB, spontaneous preterm birth.

**Fig. 5:**
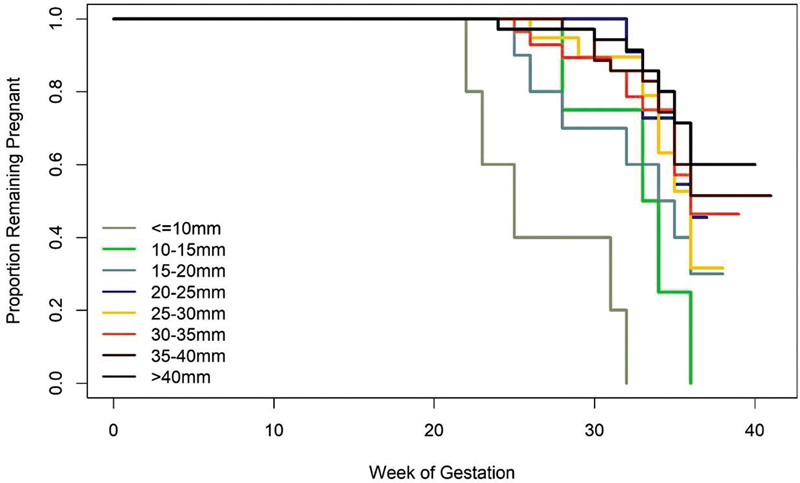
The Kaplan-Meyer survival analysis for sPTB considering different ranges of cervical length.

The best cutoff points to predict sPTB at < 34 and < 28 weeks were 21.9 and 19.95 mm, respectively. The Kaplan-Meyer survival analysis demonstrated an association between extremely severe (< 28 weeks), severe (> 28–< 34 weeks) and late sPTB (> 34–< 37 weeks), as well as CL ≤ 20 mm ( Fig. 5 ).

## Discussion

Our study provides Brazilian CL distribution curves from 18 to 22 + 6 weeks in asymptomatic twin gestations. The CL 10 ^th^ percentile was 17.8 mm and when CL was ≤ 20 mm, there was an association with extremely severe, severe, and early sPTB. However, CL was a poor predictor for sPTB in twin gestations and CL measurement by TVU did not present good performance as a screening test for spontaneous PTB.

The mean CL identified in our study and the PTB rate are very similar to previous Brazilian studies focused on twin gestations. A prospective cohort involving 341 patients with CL measurement between 18 and 21 weeks, described CL mean (31.95–33.46 mm) with 68.2% of PTB < 37 weeks. 14 However, when compared with an Italian cohort study that involved 904 twin gestations with a slightly higher CL median (35.4 vs. 38 mm), our study presented a considerably higher incidence of PTB (PTB < 32 weeks, 14.6 vs. 8.3%), which raises the possibility that other factors could be more important to influence the preterm delivery rate in twins, especially when we consider different populations. 15

Thus, before defining what is a short cervix (and its association with sPTB) in a Brazilian twin population it is crucial to know the CL distribution curve in this specific subset of women. If we consider short cervix as CL under P10, we demonstrated that in the Brazilian population CL ≤ 25 mm is not the best cutoff value for twin gestations. This way, a more interesting cutoff point would be CL ≤ 20 mm, since it is very close to P10 and demonstrated a clear association with early sPTB in the Kaplan-Meier curves.

Differently from a singleton gestation, where the maternal sociodemographic characteristics may influence the incidence of sPTB, 16 in our study, only previous PTB and funneling at measurement presented as risk factors for sPTB in twins. Our findings reinforce that, in twin gestations, maternal baseline characteristics do not influence prediction for sPTB. Additionally, the literature shows that combining CL and maternal characteristics does not seem to be the solution to increase sensitivity for screening. 17 18

Implementing a screening test for prediction is the first step for prevention, offering possible therapies when risk factors are present. However, treatments such as progesterone and cerclage used for preventing sPTB in singleton with short cervix do not demonstrate promising results in twins. 10 19 Considering the possibility to predict sPTB and administer antenatal corticosteroids, routine CL measurement did not affect the rate of twins born before 34 weeks that received lung maturation intervention. 20 In our study CL measurement by TVU in mid-trimester to predict sPTB < 37 and sPTB < 34 weeks had a poor performance and this finding was very similar to a previous Brazilian cohort that identified an AUC of 0.64 (95% CI: 0.53–0.75) for sPTB < 34. 21 Thus, considering the lack of effective interventions for preventing PTB in twins, a routine CL measurement may not improve perinatal outcomes. 22 It could also increase hospital admission rates for false labor and antepartum length of stay, leading to stress and anxiety among patients and family. Moreover, it could potentially lead to unnecessary and risky interventions, such as prescription of tocolytic drugs and bed rest. 23

A strength of our study is that our sample was composed by women from 17 different settings in Brazil, involving diverse population characteristics that can be found in a country with a continental territory.

One limitation is that almost all women with CL ≤ 30 mm received progesterone 200 mg/day and part of them also received a cervical pessary, which could have influenced the PTB's final result or even postponed PTB. However, the last studies did not show these two interventions as capable of causing a significant reduction in PTB for twins. 10 23 Also, considering that most of the participating centers were reference for high-risk pregnancies, it is possible that our distribution curve tended toward shorter CLs.

Finally, as CL does not show a good performance to predict PTB, and the available treatments for PTB in twins do not show a clear benefit, we believe that a universal screening program for twin gestation in Brazil, considering a panel with limited resources, would not be helpful or economically viable. When treatments for these high-risk populations show good efficacy, maybe a screening and treatment strategy could be justified.

## Conclusion

A cutoff point of CL ≤ 20 mm can be interesting to identify short cervix in Brazilian twin pregnancies. However, in Brazilian asymptomatic twin pregnancies, CL does not show a good performance to predict PTB. Furthermore, the available treatments for PTB in twins do not support a CL screening program in Brazil.

## References

[1] Esteves-Pereira A P da Cunha A JLA Nakamura-Pereira M Twin pregnancy and perinatal outcomes: Data from ‘Birth in Brazil Study’ PLoS One 2021 16 01 e0245152 10.1371/journal.pone.0245152 33428660 PMC7799786

[2] Santana D S Surita F G Cecatti J G Multiple pregnancy: epidemiology and association with maternal and perinatal morbidity Rev Bras Ginecol Obstet 2018 40 09 554 562 10.1055/s-0038-1668117 30231294 PMC10316907

[3] Collins J Global epidemiology of multiple birth Reprod Biomed Online 2007 15 03 45 52 10.1016/s1472-6483(10)62251-1 18598609

[4] Brazilian Multicentre Study on Preterm Birth study group Passini R Jr Cecatti J G Lajos G J Brazilian multicentre study on preterm birth (EMIP): prevalence and factors associated with spontaneous preterm birth PLoS One 2014 9 10 e109069 10.1371/journal.pone.0109069 25299699 PMC4192080

[5] The Preterm SAMBA study group Souza R T Costa M L Mayrink J Clinical and epidemiological factors associated with spontaneous preterm birth: a multicentre cohort of low risk nulliparous women Sci Rep 2020 10 01 855 10.1038/s41598-020-57810-4 31965004 PMC6972868

[6] Gudicha D W Romero R Kabiri D Personalized assessment of cervical length improves prediction of spontaneous preterm birth: a standard and a percentile calculator Am J Obstet Gynecol 2021 224 03 2880 2.88E19 10.1016/j.ajog.2020.09.002 PMC7914140 32918893

[7] Cruz-Melguizo S San-Frutos L Martínez-Payo C Cervical pessary compared with vaginal progesterone for preventing early preterm birth: a randomized controlled trial Obstet Gynecol 2018 132 04 907 915 10.1097/AOG.0000000000002884 30204689

[8] Romero R Conde-Agudelo A Da Fonseca E Vaginal progesterone for preventing preterm birth and adverse perinatal outcomes in singleton gestations with a short cervix: a meta-analysis of individual patient data Am J Obstet Gynecol 2018 218 02 161 180 10.1016/j.ajog.2017.11.576 29157866 PMC5987201

[9] National Institute of Child Health and Human Development Maternal-Fetal Medicine Units Network Goldenberg R L Iams J D Miodovnik M The preterm prediction study: risk factors in twin gestations Am J Obstet Gynecol 1996 175(4 Pt 1):1047 1053 10.1016/s0002-9378(96)80051-2 8885774

[10] D'Antonio F Berghella V Di Mascio D Role of progesterone, cerclage and pessary in preventing preterm birth in twin pregnancies: A systematic review and network meta-analysis Eur J Obstet Gynecol Reprod Biol 2021 261 166 177 10.1016/j.ejogrb.2021.04.023 33946019

[11] Brock C Moroz L Hermans F Gyamfi-Bannerman C 554: distribution of transvaginal cervical length in an unselected cohort of twin pregnancies Am J Obstet Gynecol 2016 214(1, Suppl)S298 S299 10.1016/j.ajog.2015.10.598

[12] Qureshey E Quinones J Sarno A Rust O Comparison of management options for twin pregnancies affected by midtrimester incidental cervical shortening Obstet Gynecol 2018 131 116S 10.1097/01.AOG.0000533495.84832.0f 31878811

[13] P5 Working Group Pacagnella R C Silva T V Cecatti J G Pessary plus progesterone to prevent preterm birth in women with short cervixes: a randomized controlled trial Obstet Gynecol 2022 139 01 41 51 10.1097/AOG.0000000000004634 34856583

[14] de Oliveira L A Brizot M L Liao A W Bittar R E Francisco R P Zugaib M Prenatal administration of vaginal progesterone and frequency of uterine contractions in asymptomatic twin pregnancies Acta Obstet Gynecol Scand 2016 95 04 436 443 10.1111/aogs.12843 26669629

[15] Pagani G Stagnati V Fichera A Prefumo F Cervical length at mid-gestation in screening for preterm birth in twin pregnancy Ultrasound Obstet Gynecol 2016 48 01 56 60 10.1002/uog.15668 26250480

[16] Koullali B Oudijk M A Nijman T A Mol B W Pajkrt E Risk assessment and management to prevent preterm birth Semin Fetal Neonatal Med 2016 21 02 80 88 10.1016/j.siny.2016.01.005 26906339

[17] Biggio J R Anderson S Spontaneous preterm birth in multiples Clin Obstet Gynecol 2015 58 03 654 667 10.1097/GRF.0000000000000120 26083129

[18] To M S Fonseca E B Molina F S Cacho A M Nicolaides K H Maternal characteristics and cervical length in the prediction of spontaneous early preterm delivery in twins Am J Obstet Gynecol 2006 194 05 1360 1365 10.1016/j.ajog.2005.11.001 16647922

[19] EPPPIC Group Evaluating Progestogens for Preventing Preterm birth International Collaborative (EPPPIC): meta-analysis of individual participant data from randomised controlled trials Lancet 2021 397(10280):1183 1194 10.1016/S0140-6736(21)00217-8 33773630

[20] Marcellin L Senat M V Benachi A Regis S Cabrol D Goffinet F Impact of routine transvaginal ultrasound monitoring of cervical length in twins on administration of antenatal corticosteroids J Perinat Med 2017 45 04 471 477 10.1515/jpm-2016-0102 27442356

[21] Hofmeister C Brizot MdeL Liao A Francisco R P Zugaib M Two-stage transvaginal cervical length screening for preterm birth in twin pregnancies J Perinat Med 2010 38 05 479 484 10.1515/jpm.2010.088 20629488

[22] Gordon M C McKenna D S Stewart T L Transvaginal cervical length scans to prevent prematurity in twins: a randomized controlled trial Am J Obstet Gynecol 2016 214 02 2770 2.77E9 10.1016/j.ajog.2015.08.065 26363481

[23] Berghella V Baxter J K Hendrix N W Cervical assessment by ultrasound for preventing preterm delivery Cochrane Database Syst Rev 2013 01 CD007235 10.1002/14651858.CD007235.pub3 23440813 PMC6464944

